# Cancer patients’ awareness of extent of disease: anxiety, depression, quality of life

**DOI:** 10.1136/spcare-2022-004112

**Published:** 2023-11-24

**Authors:** Maria Fidelis Coronel Manalo, Grace Meijuan Yang, Macario Reandelar Jr, Semra Ozdemir Van Dyk, Chetna Malhotra, Eric A. Finkelstein

**Affiliations:** 1 Supportive Oncology & Palliative Care, Augusto P. Sarmiento Cancer Institute, The Medical City, Pasig City, Metro Manila, Philippines; 2 Department of Community & Family Medicine, Far Eastern University Doctor Nicanor Reyes Medical Foundation, Quezon City, Metro Manila, Philippines; 3 Division of Supportive and Palliative Care, National Cancer Centre Singapore, Singapore; 4 Lien Centre for Palliative Care, Duke-NUS Medical School, Singapore; 5 Programme for Health Services & Systems Research, Duke-NUS Medical School, Singapore; 6 Research and Biotechnology, St. Luke's Medical Center, Quezon City, Metro Manila, Philippines; 7 College of Medicine, New Era University, Quezon City, Metro Manila, Philippines

**Keywords:** quality of life, depression, prognosis, cancer, communication, cultural issues

## Abstract

**ABSTRACT:**

**Objective:**

In Asian cultures, the belief that full disclosure would harm the patient’s health would lead to non-disclosure. The study aimed to determine whether making patients aware of the extent of their disease will lead to psychological morbidity and poor quality of life (QOL).

**Methods:**

This was a cross-sectional study among 195 patients with stage 4 cancer who were aware of their cancer diagnosis at the medical oncology, radiation oncology and palliative care units at an academic cancer centre. Participants were asked about their cancer stage, treatment goal and if they prefer to know their life expectancy. They answered the 14-item Hospital Anxiety and Depression Scale and 27-item validated Functional Assessment of Cancer Therapy-General questionnaires. Determination of the association of patients’ awareness of the extent of the disease with psychological status and QOL was analysed using univariate and multivariate statistics.

**Results:**

About three-fourths of patients with cancer knew they had an advanced disease, but very few were aware that the current treatments they were taking for their cancer would not cure them. No association between awareness of the extent of the disease and psychological morbidity was found. Still, those aware of the advanced disease had significantly higher QOL scores for social well-being.

**Conclusions:**

This study revealed that physicians should not hesitate to communicate the cancer diagnosis and prognosis to patients, as the disclosure was not associated with psychological morbidity. Open communication between physicians, patients and their families on the extent of the disease could empower patients to make informed decisions about their treatment, engage in advance care planning and seek the necessary support.

**WHAT IS ALREADY KNOWN ON THIS TOPIC**

- In Southeast Asian cultures, the fear that should patients know their poor prognosis, they might become depressed, worry excessively or lose the will to live has traditionally led families to request physicians for non-disclosure of diagnosis and prognosis.
- The Asian Patient Perspectives Regarding Oncology Awareness, Care and Health studies in other Asian countries revealed that patients who were aware or unsure of their prognosis reported higher levels of anxiety and depressive symptoms.

**WHAT THIS STUDY ADDS**

- One of the most important findings in this study was the absence of an association between awareness of the extent of the disease among patients with advanced cancer and psychological morbidity.
- Contrary to what might be expected, awareness of advanced cancer was associated with higher social well-being.

**HOW THIS STUDY MIGHT AFFECT RESEARCH, PRACTICE OR POLICY**

- The results of this research could impact how doctors in this cultural context communicate with patients with cancer and allay concerns among families that sharing a cancer diagnosis and prognosis with the patient could lead to distress or worry.
- Future studies could focus on examining the effect of cultural beliefs and values, such as faith and spirituality, and social support networks on the well-being of patients with cancer.

## Introduction

 In the Southeast Asia Region, there were 1 100 037 new cases of cancer and 689 093 deaths reported by the Global Cancer Observatory in 2020.^1^ In the Philippines, cancer is the second leading cause of mortality, accounting for 10.2% of the total deaths.^2^ With the magnitude of the problem that is cancer, it is of utmost importance to manage these patients, taking into account their insight into the disease. Yet patients with advanced cancer often do not fully understand that their treatment is not meant to cure but only to palliate symptoms. Thus, informed treatment decision-making requires information about the condition and the therapeutic options. Timely prognosis communication is needed for patients to put their affairs in order and prepare for the end of life.^3^ Different races and cultures, however, differ in the amount and kind of information patients with cancer would want to have. Whether or not knowledge of the extent of their disease can lead to psychological distress or a poorer outlook in life remains uncertain.

Studies in Western cultures have shown that most patients with advanced cancer prefer to be informed about their illness.^4^ A survey by Hagerty *et al* among 126 patients with metastatic cancer showed that >95% wanted information about side effects, symptoms and treatment options. The majority wanted to know the longest survival time with treatment, 5-year survival rates and average survival.^5^ Other studies showed that half of the patients with advanced cancer were unaware of their prognosis.^6^,^8^ In the Asian Patient Perspectives Regarding Oncology Awareness, Care and Health (APPROACH) study in China, India, Sri Lanka and Vietnam, results showed that among the patients with stage 4 cancer surveyed, only 28% of the patients in the sample knew that their cancer was at an advanced stage.^9^ Advanced cancer patients overestimate how long they will live.^10^ Studies from Italy, Middle Eastern and Asian countries that explored the association between awareness of diagnosis and prognosis with psychological status and quality of life (QOL) of patients with cancer showed conflicting results. Five studies revealed that aware patients had more psychological morbidity^911^,^13^ or lower subjective QOL.^14^ However, two studies showed that aware patients had less psychological morbidity^15 16^ or improvement in health-related QOL,^16^ while five studies showed no association.^17^,^21^ In the Southeast Asian culture, non-disclosure of diagnosis and non-discussion of prognosis was reported widely in surveys of oncologists or potential patients.^22^,^25^ There was fear among the family members that should patients know their poor prognosis, they might become depressed, worry excessively or lose the will to live.^22^ In Filipino culture, many family members believed full disclosure would harm the patient’s health and often asked the doctors to withhold bad news.^26 27^ However, there was a lack of studies among patients with advanced cancer in the Philippines on diagnostic and prognostic awareness and its effect on psychological status and QOL.

Given the uncertainty of findings from previous studies, and in contrast to the traditional Filipino culture of wanting to withhold bad news, we hypothesised that being aware of the extent of the disease was not associated with psychological distress or poorer QOL. We maintained the necessity of giving complete information to patients with advanced cancer and all patients in general, which would lead to a better understanding of illness and more active participation in managing their condition. Thus, we designed this study to determine the association of awareness of advanced cancer diagnosis and prognosis with psychological morbidity and QOL. Our research findings could have significant consequences for the future of cancer communication and decision-making support for patients in this cultural context. This study has helped us recognise the importance of cultural distinctions between Western and Asian societies. Ensuring patients with advanced cancers understand their prognosis accurately is essential for treatment decision-making, advance care planning and psychological support.^28^

## Methods

### Study design

This was an analytic cross-sectional study. Information on the patient’s awareness of their disease conditions, their psychological status and QOL was collected at one point in time in one interview.

### Setting

Patients were recruited from the medical oncology, radiation oncology and palliative care units at The Medical City, Philippines, from February 2018 to October 2019. Data from the Philippines became part of a multicountry study entitled ‘APPROACH’.

### Participants

We identified patients eligible for the study as those with stage 4 solid cancer, aware of their cancer diagnosis and at least 21 years of age. Physicians from the study site identified patients who met the eligibility criteria and referred them to the study team. Patients were then consecutively approached during their hospital appointments or hospitalisation and invited to participate in the study. While participants were encouraged to answer all sections of the questionnaire completely, they had the option not to answer any question they would rather not.

### Variables, data sources and measurement

Exposure variables for this study included awareness of advanced cancer and prognosis, with a preference to know the life expectancy as a potential effect modifier. Outcome variables included psychological morbidity and QOL. Potential confounders included age, sex, marital status, education and financial capability to cover the cost of treatment.

Awareness of advanced cancer was defined from a question: “Do you know the current stage (ie, severity) of your cancer?” with options of early stage (stages 1, 2 and 3), advanced stage (stage 4) and I do not know. Those who answered ‘advanced stage’ were categorised as being aware. Those who responded otherwise were classified as unaware.

Prognostic awareness was assessed from the statement: “The current treatments you are taking for your illness will cure you”. ‘No’ responses were categorised as ‘aware of prognosis’, ‘not sure’ responses were classified as ‘not sure of prognosis’ whereas ‘yes’ responses were classified as ‘unaware of prognosis’. The question was adapted from the Cancer Care Outcomes Research and Surveillance Consortium study.^29^

Preference for knowing life expectancy was examined using the statement: “Would you like to know how long you are likely to live under various treatment options?” (eg, standard treatments like chemotherapy, radiation, surgery or supportive care alone). ‘No’ and ‘not sure’ responses were categorised as ‘prefers not to know’ whereas ‘yes, in general terms such as a few months or a few years’ and ‘yes, in specific terms such as on average 6 months’ were categorised as ‘prefers to know’. This question has been used to assess preference for knowing life expectancy among patients with cancer.^4^

Psychological morbidity was measured using the 14-item Hospital Anxiety and Depression Scale. Participants who scored 8 and above were identified as having clinically relevant anxiety and/or depressive symptoms (probable anxiety/depression). Those who scored below 8 were identified as having no anxiety and/or depression.^30^

QOL was measured using the 27-item validated Functional Assessment of Cancer Therapy-General questionnaire with four domains of health-related QOL: physical, social, emotional and functional well-being. The sum from all domain scores was also calculated.^31^ Higher scores indicated greater well-being.

All potential confounders were classified into two categories, with age as below 60 years or 60 years and above, sex as male or female, marital status as with or without a spouse, education as having reached college or up to high school and financial capacity as fairly to very well or poorly well.

### Bias

As inherent in all cross-sectional studies, temporal ambiguity is always a drawback. The generalisability of the findings could be limited due to the recruitment of patients from an academic institution, where a majority of the patients belong to a higher socio-economic status compared with the broader population in the Philippines, and the use of a non-probability sampling method.

### Study size

Being part of a multicountry study, this study used a sample size calculated from an appropriate number of subjects per country that ensured sufficient precision and power in answering the research questions while maintaining feasibility,^29^ giving a total of 200 participants.

### Statistical methods

Descriptive statistics were done on the patient’s sociodemographic profile and awareness of the extent of the disease condition. For analytical statistics, categorical variables were dichotomised and presented as counts and percentages. For missing item responses, a missing category was created for categorical responses. Determination of the association of patients’ awareness of the extent of the disease with psychological status was analysed using univariate and multivariate statistics. In the univariate analysis, the χ^2^ test was used for the psychological status outcome. Multiple logistic regression was then used in the multivariate analysis with a preference to know life expectancy interacting with awareness of the extent of the disease. With the outcome of QOL, an independent t-test and analysis of variance were used in the univariate analysis. Multiple linear regression was then made for the multivariate analysis. The level of significance was set at p=0.05. The IBM SPSS Statistics V.28 was used for the analyses.

## Results

### Participants

A total of 200 individuals were surveyed. Four participants did not have solid cancers, and one was found during the interview to be mentally incompetent. These participants were excluded, leaving a total of 195 participants for analyses. Thirty-four, 27, 2 and 1 participant opted not to answer the questions on life expectancy, prognosis, financial capability and anxiety/depression, respectively.

### Descriptive data

Respondents’ mean age was 56.4±14.46 years, and about two-thirds were female. Most were married, had a college education and all identified as Christian. It was interesting to note that about three-fourths knew that their cancer was at an advanced stage, and about half preferred to know their life expectancy, yet <5% were aware of their prognosis. A little less than three-fourths had either anxiety or depression or both (table 1).

**Table 1:** Sociodemographic and clinical profile of patients with advanced cancer

Characteristics, n=195	N (%)
Age (years)	
<60	101 (51.8)
60 and older	94 (48.2)
Sex	
Male	75 (38.5)
Female	120 (61.5)
Marital status	
With spouse	154 (79.0)
Without spouse	41 (21.0)
Education	
College and higher education	162 (83.1)
Up to high school	33 (16.9)
Financial capability to cover cost of treatment	
Fairly to very well	129 (66.8)
Poorly well	64 (33.2)
Type of patient	
Outpatient	154 (79.0)
Inpatient	41 (21.0)
Type of cancer	
Breast	43 (22.1)
Lung	38 (19.5)
Colorectal	25 (12.8)
Nasopharyngeal	17 (8.7)
Endometrial	8 (4.1)
Aware of advanced cancer	
Aware of advanced stage	146 (74.9)
Unaware of advanced stage	49 (25.1)
Prefer to know life expectancy	
Yes, in general terms	66 (41.0)
Yes, in specific terms	46 (28.6)
No	25 (15.5)
Not sure	24 (14.9)
Prognostic awareness (understanding of treatment intent)	
Aware of prognosis	5 (3.0)
Unaware of prognosis	126 (75.0)
Not sure about prognosis	37 (22.0)
Anxiety/Depression	
Yes	137 (70.6)
No	57 (29.4)

### Main results

We examined the association of awareness of advanced disease and prognosis with anxiety and/or depression. Univariate analysis showed that only awareness of the prognosis had statistically significant results. Those not aware of the prognosis and those unsure were more likely to have anxiety/depression than those aware. Interestingly, with marital status, those with a spouse had a significantly higher proportion of anxiety/depression compared with those without a spouse (table 2).

**Table 2:** Univariate analysis of the association between awareness of the extent of disease and psychological status of patients with advanced cancer

Characteristics	Anxiety/Depression n (%)	P value
Age (years)		0.610
<60	69 (69.0)	
60 and older	68 (72.3)	
Sex		0.755
Male	52 (69.3)	
Female	85 (71.4)	
Marital status		0.022
With spouse	114 (74.5)	
Without spouse	23 (51.6)	
Education		0.334
College and higher education	116 (72.0)	
Up to high school	21 (63.6)	
Financial capability to cover cost of treatment		0.630
Fairly to very well	93 (72.1)	
Poorly well	44 (68.8)	
Aware of advanced cancer		0.111
Aware of advanced stage	98 (67.6)	
Unaware of advanced stage	39 (79.6)	
Prefer to know life expectancy		0.058
Yes	112 (75.9)	
No	49 (61.2)	
Prognostic awareness (understanding of treatment intent)		0.040
Aware of prognosis	1 (20.0)	
Unaware of prognosis	91 (72.2)	
Not sure about prognosis	27 (73.0)	

In the multivariate analysis, we did not find any statistically significant findings in the association between awareness of the extent of the disease and anxiety/depression. Interaction of the awareness of advanced cancer with a preference to know life expectancy did not likewise show significant findings. As in the univariate analysis, marital status was significantly associated with anxiety/depression. Holding all other variables constant, those with a spouse were more likely to have anxiety/depression compared with their counterparts (table 3).

**Table 3:** Multivariate analysis of the association between awareness of the extent of disease and psychological status of patients with advanced cancer

Characteristics	Anxiety/Depression OR (95% CI)	P value
Age (years)		0.375
<60	0.71 (0.33 to 1.52)	
60 and older	*--	
Sex		0.223
Male	0.61 (0.28 to 1.35)	
Female	*--	
Marital status		0.027
With spouse	2.82 (1.13 to 7.04)	
Without spouse	*--	
Education		0.053
College and higher education	2.53 (0.99 to 6.50)	
Up to high school	*--	
Financial capability to cover cost of treatment		0.279
Fairly to very well	1.55 (0.70 to 3.45)	
Poorly well	*--	
Aware of advanced cancer		0.760
Aware of advanced stage	0.76 (0.18 to 3.58)	
Unaware of advanced stage	*--	
Prefer to know life expectancy		0.076
Yes	5.10 (0.84 to 30.85)	
No	*--	
Prognostic awareness (understanding of treatment intent)		0.429
Aware of prognosis	7.00 (0.36 to 134.84)	0.197
Unaware of prognosis	7.35 (0.35 to 154.79)	0.200
Not sure about prognosis	*--	
Aware of advanced disease*—prefer to know life expectancy	0.34 (0.05 to 2.53)	0.293

* -- Reference.

Lastly, in the association of the awareness of the extent of the disease and QOL, we found in the univariate analysis that those who were aware of the advanced disease and of the prognosis had significantly higher social well-being scores than their counterparts. There were, however, no statistically significant findings in the total well-being scores. We then did a multivariate analysis for social well-being scores, and indeed, we found out that holding all other variables constant, results showed an average of 2.71 increase in the scores among those aware of advanced disease compared with those unaware. Results also showed that married patients had a decrease in their social well-being scores compared with those without a spouse (tables4  5).

**Table 4:** Univariate analysis of the association between awareness of the extent of disease and quality of life

Awareness of the extent of the cancer	FACT-G physical well-being Mean±SD	FACT-G social well-being Mean±SD	FACT-G emotional well-being Mean±SD	FACT-G functional well-being Mean±SD	FACT-G total Mean±SD
Aware of advanced cancer†					
Aware of advanced stage	14.5±5.94	20.3±4.12*	15.8±4.43	15.6±5.04	66.1±14.24
Unaware of advanced stage	13.2±5.83	17.6±4.56*	16.8±3.70	15.9±3.91	63.4±12.15
Prognostic awareness‡					
Aware of prognosis	15.8±8.14	23.5±5.72*	17.4±5.37	16.2±7.19	72.9±22.68
Unaware of prognosis	14.5±6.01	19.2±4.18*	16.5±4.12	15.2±4.74	65.4±13.61
Not sure about prognosis	13.5±5.62	20.6±4.93	15.1±4.64	17.4±4.54	66.6±13.05

* significant at p value <0.05.

† Independent t-test.

‡ Analysis of variance.

FACT-G, 27-item validated Functional Assessment of Cancer Therapy-General.

**Table 5:** Multivariate analysis of the association between awareness of the extent of disease and social well-being quality of life scores

Characteristics	FACT-G social well-being Beta-coefficient (95% CI)	P value*
Younger than 60 vs 60 and above	1.34 (0.08 to 2.61)	0.037
Male versus female	0.17 (−1.14 to 1.47)	0.803
Reached college versus did not reach	0.16 (−1.53 to 1.85)	0.855
Very well-off versus poorly well-off	−0.48 (−1.84 to 0.89)	0.491
With spouse versus without	**−2.43 (−4.06 to 0.81)**	**0.004**
Aware of advanced cancer versus unaware	2.71 (1.27 to 4.16)	<0.001
Aware of prognosis versus unaware versus not sure	0.62 (−0.79 to 2.03)	0.618

Bold letters indiciate that this result is significant.

* Statistical test—multiple linear regression.

FACT-G, 27-item validated Functional Assessment of Cancer Therapy-General.

## Discussion

### Key results

We hypothesised that being aware of the extent of the disease was not associated with psychological distress or lower QOL. After adjusting for all confounders, we have confirmed our hypothesis for the psychological distress, that is, there was no association between awareness of the extent of the disease and psychological morbidity. However, awareness of advanced cancer was associated with higher social well-being scores for the QOL.

### Limitations

Our study has limitations. First, we conducted this study in an academic cancer centre in a relatively homogenous sample of patients with higher socioeconomic and educational levels than the average Filipino. It is possible that the results reported may not be generalisable to patients with cancer throughout the Philippines and other countries in the region. Second, we lacked information about patient-clinician communication. We did not have access to physician-reported data on whether and to what extent the patient was informed of their stage 4 disease. From our anecdotal clinical experience, in our local context, doctors nowadays generally tell patients that they have cancer as they explain the histopathology result to the patients. Still, doctors do not generally tell them the stage of their cancer unless the patient or family asks about it. Third, we did not look into the possibility of denial, which could affect awareness of the extent of the disease. Fourth, there may also be other unmeasured confounders that may be associated with psychological state and QOL in patients with cancer. We made no adjustment of these potential unmeasured variables in our analysis. Finally, we can only infer association with our cross-sectional design since our data were collected only at one point, limiting our ability to determine how these relationships changed over time.

### Comparison with other studies

Our study demonstrated that the majority of patients with advanced cancer, about 75%, were aware of their actual stage. This result was consistent with two prior local studies that showed preferences for disclosure among Filipino patients with cancer.^32 33^ This, however, was contrary to the APPROACH study, where only 28% of patients with stage 4 cancer knew their cancer was at an advanced stage.^9^ In recent years, cancer care communication preferences of Asians, including Filipino patients, have gradually shifted towards more open communication, partly due to the effects of globalisation of liberal values (freedom, autonomy and choice).^34^ However, only a small proportion of our patients (3%) were prognostically aware (ie, aware that the current treatments they are taking for their cancer will not cure them). This is below the 56.2% (median=66%) of prognostically aware patients from studies conducted in Asia from a recent systematic review by Ng and Ozdemir.^35^ In this way, our results were in keeping with the belief of some Filipino physicians^33^ and family members^27^ that disclosure of a poor prognosis could diminish the patient’s hope and will to fight the illness. It also corroborated the traditional practice of family caregivers requesting physicians to conceal the stage of cancer and its prognosis from the patient due to their belief that only God can decide a person’s fate.^27^ Language barriers, health literacy levels, the complexity of medical information or the presence of denial, which in patients with cancer was noted to have a prevalence of 4%–47%,^36^ could also hinder accurate understanding, leading to misconceptions or incomplete awareness of diagnosis and prognosis. One of the most important findings in this study was the absence of an association between awareness of the extent of the disease among patients with advanced cancer and psychological morbidity. Our results differed from the APPROACH studies, where patients who were aware or unsure of their prognosis reported higher levels of anxiety and depressive symptoms.^9^ Filipino patients often relied on various coping mechanisms, including the support of their families, religious or spiritual practices and resilience. The effectiveness of these coping mechanisms might influence how patients would manage any possible psychological distress associated with their prognosis. Additionally, our study revealed that awareness of the advanced cancer stage was associated with better social and family well-being. This finding was similar to some studies that those who were well-supported socially were more able to handle information about disease prognosis^37^ and that prognostic awareness facilitated open conversations and better social well-being.^38^

An incidental finding we noted in this study was that married patients had higher chances of having anxiety/depression compared with patients without a spouse. Our results contrasted with a study that married patients with cancer with high partner support reported significantly lower levels of psychological distress.^39^ Among Filipinos, while social support from a spouse could be beneficial, it could also amplify the impact of a cancer diagnosis on a married patient’s mental health. The patient might feel compelled to mask their distress to provide emotional support to their spouse, which could lead to suppressed emotions and heightened psychological distress. Married patients with cancer might experience heightened anxiety and distress due to their inability to fulfil their spouse’s needs and their role in the family, participate in family functions and work and support their family.^40^

### Implications

This study has shown that physicians should not hesitate to communicate the advanced cancer diagnosis and prognosis to patients since disclosure was not associated with psychological morbidity. On the contrary, awareness of advanced cancer was associated with higher social well-being. Thus, discussing the extent of the disease sensitively and compassionately could empower patients to make informed decisions about their treatment and advance care planning. This could also allow patients to communicate openly and honestly and spend quality time with their families. The findings may prompt further research exploration into other factors that influence the psychosocial outcomes and QOL of patients with advanced cancer in the Philippines. Future studies could focus on examining the impact of cultural beliefs and values, such as faith and spirituality, and social support networks on the well-being of patients with cancer.

## Conclusion

We have demonstrated that awareness of the extent of the disease was not associated with psychological morbidity. Instead, those aware of the extent of the disease had even higher social being scores compared with those unaware. It is worth mentioning the incidental findings that married individuals had a significantly higher prevalence of anxiety/depression and had lower scores in social well-being than those without a spouse.

## Data Availability

All data relevant to the study are included in the article.
